# Bridging the gap: a cross-sectional study on biomedical waste management education and compliance in dental institutions of Delhi National Capital Region

**DOI:** 10.3205/dgkh000553

**Published:** 2025-05-26

**Authors:** Shakila Mahesh, Kruthiventi Hemalata, Ramya Shanta, Urvi Vashistha, Kavya Krishnakumar, Samridhi Arora, Alpa Gupta

**Affiliations:** 1 Department of Microbiology, Manav Rachna Dental College, School of Dental Sciences, MRIIRS, Faridabad, India; 2 Manav Rachna Dental College, School of Dental Sciences, MRIIRS, Faridabad, India; 3 Department of endodontics and conservative dentistry, School of Dental Sciences, Manav Rachna International Institute of Research and Studies, Haryana, India

**Keywords:** biomedical waste management, dental college, undergraduates, post-graduates, faculty, knowledge, practice, attitude

## Abstract

**Introduction and method:**

Biomedical waste management (BMWM) ensures the safe handling, segregation, and disposal of healthcare waste from hospitals, clinics, and laboratories. It reduces infection risks, protects public health, and promotes environmental sustainability, benefiting healthcare workers, patients, and communities. The purpose of this study was to assess BMWM knowledge, attitudes, and practices among healthcare professionals using a structured questionnaire. Data were analyzed by participants' educational levels.

**Results:**

The study included 180 voluntary participants: 28 faculty members, 33 postgraduate students from various disciplines, and 119 undergraduate students from a dental college. 41.7% correctly identified black as the color code for general waste, and 73.8% knew needle syringes require puncture-resistant bins. Awareness of sharps containers and blood waste incineration (53.9%) was moderate. While 68.9% supported BMWM in undergraduate curricula, 91.1% stressed the importance of segregation. Autoclaving was used by 71.7% for sterilization, and 47.8% used special containers for lab samples. Faculty members had the highest knowledge scores.

**Discussion:**

The study revealed moderate understanding of BMWM, with 55–60% showing full comprehension. Mercury disposal awareness (42.8%) was higher due to coverage in the dental curriculum. Collaboration among healthcare professionals and improved training are vital for effective BMWM practices.

## Zusammenfassung

**Einleitung:**

Das Management biomedizinischer Abfälle (MBMA) gewährleistet die sichere Handhabung, Trennung und Entsorgung von medizinischen Abfällen aus Krankenhäusern, Kliniken und Laboren. Es reduziert das Infektionsrisiko, schützt die öffentliche Gesundheit und fördert die ökologische Nachhaltigkeit, was sowohl dem medizinischem Personal als auch Patienten und der Gesellschaft zugutekommt.

**Methode:**

Mit Hilfe eines strukturierten Fragebogens wurden Wissen, Einstellungen und Praktiken von medizinischem Fachpersonal im Hinblick auf das MBMA bewertet. Die Daten wurden entsprechend dem Bildungsniveau der Teilnehmer analysiert.

**Ergebnisse:**

180 freiwillige Teilnehmer, darunter 28 Fakultätsmitglieder, 33 Postgraduierte verschiedener Fachrichtungen und 119 Studenten einer zahnmedizinischen Hochschule, nahmen an der Befragung teil. 41,7% identifizierten die Farbe Schwarz korrekt als Kennzeichnung für allgemeinen Abfall. 73,8% wussten, dass Injektionsnadeln in stichfesten Behältern entsorgt werden müssen. Das Bewusstsein zur Entsorgung spitzer Gegenstände und die Verbrennung von Blutabfällen (53,9%) waren moderat. Während 68,9% die Integration von BMWM in die vorklinische Ausbildung befürworteten, betonten 91,1% die Bedeutung der Abfalltrennung (p=0,003). Autoklavieren wurde von 71,7% zur Sterilisation eingesetzt, und 47,8% verwendeten spezielle Behälter für Laborproben. Das Lehrpersonal hatte das beste Wissen.

**Fazit:**

Die Studie offenbarte ein moderates Verständnis für das BMWM, wobei 55–60% vollständiges Verständnis zeigten. Das Bewusstsein für die Entsorgung von Amalgam (42,8%) war aufgrund des zahnmedizinischen Lehrplans höher. Die Zusammenarbeit zwischen medizinischen Fachkräften und verbesserte Schulungen sind entscheidend für eine effektive Umsetzung der BMWM-Praktiken.

## Introduction

In recent years, the number dental and medical colleges has increased, implying there has been a tremendous increase in the production of biomedical waste. Biomedical waste is defined as “any solid, fluid or liquid waste, including its container and any intermediate product, which is generated during diagnosis, treatment or immunization of human beings or animals, in research pertaining thereto, or in the production or testing of biological and animal wastes from slaughterhouses or any other like establishments” [1]. In broad terms, biomedical waste can be categorized into chemical, cytotoxic, general or non-hazardous, infectious, pharmaceutical, pathological, and radioactive wastes [2], [3].

The waste produced by dental clinics can be broadly divided into two categories: hazardous products and infectious material, which is more of an immediate risk for individuals if not managed properly [4]. Although dental clinics, in comparison to other medical facilities, produce less biomedical waste, it was noted that in last decade there had been a significant increase in the amount of dental solid waste. This solid waste chiefly consists of gloves, masks, and plastic barriers [5].

Dental solid wastes mostly contain non-hazardous wastes. The hazardous waste produced is a small component, comprising amalgam, X-ray films, lead foils, and lead aprons in major quantities, while unused chemicals and drugs are produced in minor amounts. As amalgam consists of almost 3% mercury, it leads to environmental concerns due to itstoxic nature [6]. When the sewer system in a metropolitan city (e.g., Seattle) was scanned, it was found that about 14% of the mercury present originated from dental practices/clinics [7].

The Indian government has developed many guidelines for proper waste management from the healthcare sector, including the Atomic Energy Act of 1962 for radioactive wastes, Manufacture, Storage and Import of Hazardous Chemicals Rules in 1989 for hazardous chemicals, Municipal Solid Waste Rules in 2000 for solid waste, Batteries (Management and Handling) Rules in 2001 for handling lead-containing waste, Biomedical waste (Management and Handling) Rules in 1998, Biomedical Waste Management in 2016, an illustrated guide to Biomedical Waste Management amended in 2018 and 2019, and more [8], [9]. Table 1 (Tab. 1) provides information regarding common biomedical wastes, their categorization, and treatment and disposal options [8].

Regardless of such guidelines given by the government, implementing them has been a challenge. The execution is chiefly impacted due to inadequate training of the staff dealing with biomedical waste, technology, proper transport, and economic difficulties [10]. A study found that almost 50% of the dental practitioners are not aware of the laws and regulations of biomedical waste management [11]. The study conducted by Sudhaker et al. [12] showed that the most common problem practitioners encounter while managing biomedical waste is the lack of appropriate waste management services, and nearly 17% lack information regarding the same. 

Hence, the aim of this study is to assess the knowledge, attitude, and practice of biomedical waste management among students and faculty members of the dental college in Delhi National Capital Region (NCR).

## Methods

A study was conducted using a questionnaire with closed-ended questions. The questionnaire was designed to evaluate the knowledge, attitude and practice of the participants regarding biomedical waste management. 

### Study population and sample selection 

The study population consisted of 180 voluntary participants, including 28 faculty members, 33 postgraduate students from various disciplines, and 119 undergraduate students from a dental college in the Delhi NCR region. A total of 250 individuals were contacted and 180 agreed to participate in the survey. The response rate was 72%. Convenience sampling was used to select participants. Inclusion criteria were limited to undergraduate and postgraduate students, as well as faculty members, from the dental college in Delhi NCR. Individuals not affiliated with the dental college were excluded from the study.

### Study design 

The questionnaire was shared with university students and faculty through Google Forms, enabling data collection. Consent was obtained from participants before they began filling out the questionnaire.

### Data collection 

The questionnaire, based on multiple previous studies and validated by three subject experts [13], [14], [15], [16], [17], [18], included 59 questions organized into three main categories:

1. knowledge (19 questions),
2. attitude (20 questions), and
3. practice (20 questions).

It was distributed using Google Forms.

### Data analysis 

The percentage of correct and incorrect responses was calculated for knowledge-based questions. For the attitude section, responses were rated on a 5-point Likert scale from 1 to 4. Scores for each category were then totaled. The data collected was entered into an Excel sheet for organization and further analysis, with statistical analysis conducted using SPSS software.

## Results

The analysis of Biomedical Waste Management (BMWM) knowledge among faculty, postgraduate, and undergraduate students reveals significant differences in awareness across various areas. Undergraduates showed notable gaps in their understanding, particularly in training and knowledge of hazards. A higher proportion of undergraduates (46.1%) reported no training in BMWM compared to faculty (8.9%) and postgraduates (8.3%) (p=0.029). Similarly, 24.4% of undergraduates were unaware of hazards associated with BMWM, whereas only 1.1% of faculty and 4.4% of postgraduates lacked this awareness (p=0.006). Faculty and postgraduates demonstrated better knowledge of the biohazard symbol, with all faculty members reporting awareness, while 15% of undergraduates and 1.2% of postgraduates were unaware (p=0.006) (Table 2 (Tab. 2)).

The understanding of color-coding systems for waste segregation also varied significantly, with faculty and postgraduates showing complete awareness, while 16.1% of undergraduates were not aware (p=0.026). Overall, faculty and postgraduates exhibited higher levels of BMWM knowledge, likely due to their professional training and experience, while undergraduates consistently showed lower awareness (Table 2 (Tab. 2)).

Regarding waste disposal practices, significant differences were observed in the disposal of blood-contaminated items, with 61.7% of respondents correctly identifying yellow bags for disposal. However, undergraduates (37.8%) lagged behind faculty (10%) and postgraduates (13.9%) in adhering to correct disposal methods. For pharmaceutical waste, 40.6% of all groups selected the correct black bag, but 22.8% chose the red bag, indicating some confusion among respondents. The disposal of mercury and sharps also revealed discrepancies, with 52.8% correctly choosing a sharp container, and 42.8% selecting an airtight plastic container for mercury (Table 2 (Tab. 2)).

Knowledge about storage durations and segregation procedures was generally good, with 58.9% of participants correctly identifying 48 hours as the maximum duration for waste storage. However, only 36.7% of undergraduates selected this option, indicating some knowledge gaps. After blood exposure, proper steps help minimize infection risk. Washing with soap and water was reported by 21.1% of participants. Antiseptic application for needlestick injuries was practiced by 34.4%, while eye irrigation and skin flushing were performed in 31.7% and 12.8% of participants, respectively. These measures are crucial in reducing blood borne pathogen risks. (Table 2 (Tab. 2)).

The study highlights the need for targeted interventions for undergraduates to improve their BMWM knowledge and practices. Faculty and postgraduates demonstrated better understanding, but continuing education is essential for all groups to ensure proper BMWM compliance and safety.

On analyzing the response to attitude questions based on education level in Table 3 (Tab. 3), it was found that (68.9%) of participants significantly (p=0.001) strongly agreed that BMWM should be part of the UG curriculum. With a significant number of participants disagreed that BMWM is the responsibility of government (p=0.001). 91.1% of the participants agreed that segregation is necessary before waste disposal (p=0.003). 38.9% of the participants strongly disagreed that BMWM requires team work (p=0.002).

On analyzing the response to practice questions based on education (Table 4 (Tab. 4)), it was found that (71.7%) of the participants answered that autoclave was the method of sterilization used. 47.8% of the participants responded that they use a special container for packing gypsum-based casts for transport to laboratory.

It was observed that the faculty members have the highest knowledge, followed by postgraduate and undergraduate students (Table 4 (Tab. 4) and Table 5 (Tab. 5)).

The significant difference suggests variation in knowledge levels about BMWM among faculty, postgraduates, and undergraduates. The faculty have the highest mean score, reflecting more knowledge and experience. Undergraduates have the lowest mean score, given their limited exposure.

**Figure 1:**
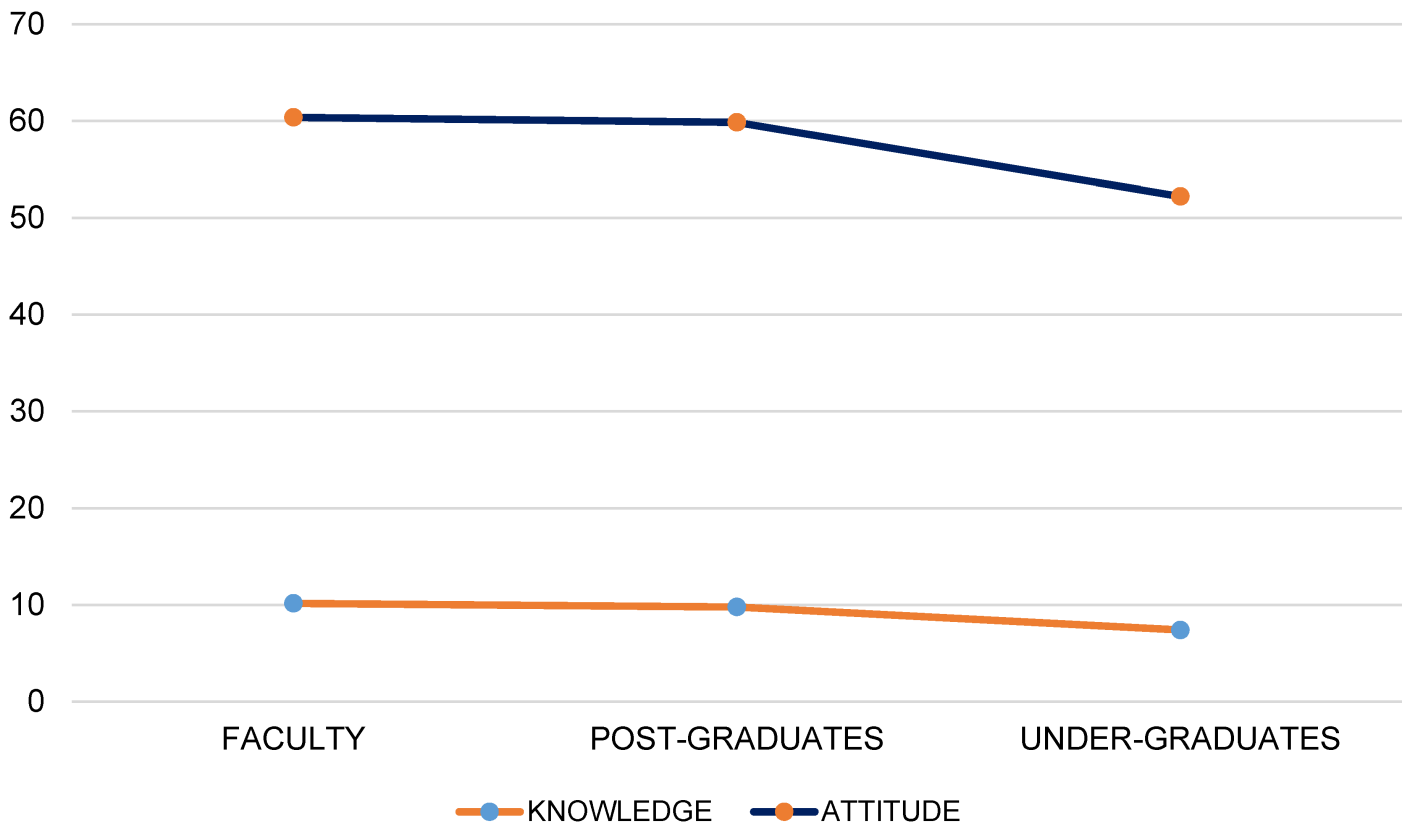
Depiction of the scores of each category, i.e., knowledge and attitude, scores of faculties, post-graduates and undergraduates

Figure 1 (Fig. 1) depicts the scores of each category, i.e., knowledge and attitude, scores of faculties, post-graduates and undergraduates. 

## Discussion

Biomedical waste management is a critical aspect of healthcare facilities and research institutions, as it involves the proper handling, treatment, and disposal of potentially hazardous materials. Improper management of biomedical waste can pose significant risks to human health and the environment, including the spread of infectious diseases, environmental pollution, and potential injuries to workers and the general public.

One of the key challenges in biomedical waste management is the segregation and classification of waste at the point of generation. Several studies have highlighted the importance of proper segregation and the need for training and awareness among healthcare workers and staff [19], [20], [21]. 

Healthcare waste poses serious infection and injury risks, with improper handling impacting health and the environment. In this study, 81.7% of faculty, undergraduates, and postgraduates knew the color coding (p<0.02), 59% understood that biomedical waste (BMW) should not be stored longer than 48 hours, and 53% recognized the need for puncture-proof containers for sharps (p<0.001) (Table 1 (Tab. 1)). Another study by Mathur et al. [20] in Allahabad (India) hospitals found that doctors, nurses, and lab technicians had higher awareness of waste management protocols than did sanitary staff, with low injury reporting across all groups. Dixit et al. [21] in Uttar Pradesh healthcare facilities showed that guidelines were available primarily in tertiary (93%) and secondary (51.5%) care centers, and that doctors had a significantly higher awareness of hazards, prevention, and waste handling than did nurses (p<0.001). Additionally, 46.1% in the present study agreed, and 39.4% strongly agreed, that training is crucial for effective waste management (Table 2 (Tab. 2) and Table 3 (Tab. 3)).

The treatment and disposal of biomedical waste are also crucial components of effective waste management. Various treatment methods have been explored, including autoclaving, chemical disinfection, and incineration. However, each method has its advantages and limitations, and the choice of treatment method depends on factors such as the type of waste, available resources, and local regulations [22], [23].

In addition to treatment methods, the transportation and final disposal of biomedical waste are equally important. Studies have highlighted the need for proper containment, labelling, and transportation protocols to minimize the risk of exposure and environmental contamination [22], [23]. In the present study, 54% agreed that blood waste should be incinerated, 36% new contaminated plastic waste containing culture, clinical specimens, and infected catheters need to be autoclaved (Table 2 ). Two studies have highlighted the positive and negative impact of two methods of waste disposal, i.e., is autoclaving and incineration [22], [23]. In a study by Sohrab Hossain et al. [22], steam autoclaving was tested as an alternative to sterilization by incineration to kill bacteria in clinical solid waste, examining contact times (0–60 min) and temperatures (111–131°C) under automated steam pressure. Results showed bacterial reduction with higher temperatures and longer contact times. Optimal conditions were 121°C for 15 minutes for gram-negative bacteria and 121°C for 60 minutes or 131°C for 30 minutes for gram-positive bacteria. However, bacterial re-growth began two days post-sterilization, suggesting the steam autoclave is not a viable alternative to incineration for clinical waste management [22].

Medical waste requires special disposal methods before landfilling, with infected waste needing treatment. Incineration, a traditional method, faces objections due to emissions of CO_2_, CO, and carcinogens such as dioxins and furans from incomplete PVC combustion. Autoclaving, a newer, wet disinfection method, was studied in Isfahan hospitals using TST and spore tests, which confirmed successful treatment. Incinerator emissions showed high CO levels and under 99.5% efficacy, below Iran’s waste management standards. Autoclaves proved safer, with no needle-stick injuries during waste handling, and had lower maintenance costs despite higher initial investments. Due to poor incinerator performance and limited anatomical waste, a combination of centralized and mobile autoclaves is recommended for waste treatment in Isfahan. Prioritizing waste management training for hospital staff is essential to minimize waste and improve separation practices [23].

Several countries have implemented regulations and guidelines for biomedical waste management, such as the Biomedical Waste Management Rules in India and the Environmental Protection Agency [EPA] regulations in the United States. However, compliance with these regulations remains challenging, particularly in low-resource settings [21], [24]. The study by Almuneef & Memish (2003) demonstrated that implementing an effective medical waste management plan led to a 58% reduction in medical waste and a 50% decrease in total financial costs. This included savings in fuel, labour, and maintenance expenses, while also reducing environmental pollution and health risks. The findings highlighted that proper waste management is both feasible and beneficial for healthcare facilities [25].

Our study reveals the current understanding of dental students regarding the proper disposal of biomedical waste. Notably, a significant portion of respondents expressed a basic understanding of the importance of biomedical waste management education and the need to follow regulations. Approximately 55–60% of responses reflected a comprehensive grasp of these key aspects. Additionally, 62.2% strongly agreed about the importance of precautions against needle-stick injuries (p=0.005), and 66.7% strongly supported waste segregation practices (p=0.003), as shown in Table 3 (Tab. 3). Furthermore, 46.1% agreed and 39.4% strongly agreed that they require further training in BMWM (p=0.05). This finding aligns with research conducted among dental students in Nepal [26]. In their study most participants (91.82%) showed a positive attitude toward safe biomedical waste management. While 83.1% to 98.9% of students had a favourable view of safe practices, over half were unaware of Nepal’s official guidelines [26]. Awareness of hospital waste disposal techniques varied widely (29.9% to 79.8%), suggesting a need for stricter protocols. Responses on the health risks of improper waste management were high across colleges, ranging from 93.3% to 98.9% [26].

Effective biomedical waste management requires a multidisciplinary approach involving healthcare professionals, waste management experts, policymakers, and the general public. Continuous training, awareness campaigns, and implementing best practices are crucial for minimizing the risks associated with biomedical waste [26], [27], [28].

In the current study, only 48% of all participants (including faculty, undergraduate and postgraduates) had knowledge of proper mercury disposal, indicating that excess mercury should be placed in airtight containers (Table 2 (Tab. 2)). This problem no longer exists in the European Union because amalgam fillings have been banned since January 1, 2025. Similarly, Singh et al. [29] found that 63.7% of dentists were unaware of biomedical waste categories, with just 31.9% recognizing outdated drugs as cytotoxic waste. For developer and fixer solutions, 45% discharged them into the sewer, 49.4% diluted them first, and only 5.6% returned them to the supplier, while 40.6% disposed of silver amalgam in common bins. 

It is interesting to note that a Nigerian study [30], reporting a 90.9% response rate, found 95.7% of specialists and 74.5% of general dentists to support the safety of amalgam fillings. Most dentists (81%) opposed an amalgam ban, with 84.3% not recommending alternatives, thus showing a broad acceptance. The study highlighted the need for increased awareness and education on amalgam safety and proper handling practices.

The current practice-based questions (Table 4 ) provided insights into the waste management behaviours of dental students. Among the findings, 83.3% considered all patients potentially infectious (p=0.014), and 71.7% identified autoclaving as the best method of sterilization (p=0.001). Additionally, 47.8% used special containers for packing gypsum casts (p=0.001), and 86.7% follow post-exposure prophylaxis (PEP) after a needle injury (p=0.001). Of the respondents, 67.3% believe in using personal protective equipment (PPE), while 90.6% support BMWM at the point of patient care. Furthermore, 79.4% report incidents, 60.6% use needle cutters before disposal, 73.3% practice handwashing after handling biomedical waste, and 85% label containers before filling them with waste. A similar cross-sectional survey was conducted among 222 dental undergraduates and interns at the Dental Institute, Rajendra Institute of Medical Sciences, Ranchi, India [31]. The study assessed participants’ knowledge, attitudes, and practices (KAP) on sterilization and disinfection before and after educational lectures using a structured questionnaire. All 182 respondents acknowledged the importance of sterilization in dental procedures. Compliance with hand hygiene was high (100%), and 78.8% were aware of autoclave sterilization. The study assessed knowledge of Biomedical Waste (BMW) management across different education levels. Faculty members had the highest mean knowledge score (10.14±1.99), followed by postgraduate students (9.76±2.06), while undergraduate students had the lowest score (7.38±2.80). The overall mean knowledge score was 8.24±2.83, with a significant difference (p=0.001), indicating that higher education levels were associated with better knowledge of BMW management.

Another study with 186 participants assessed knowledge on biomedical waste management across five areas: laws, waste handling, dental waste categories, disposal hazards, and specific techniques. Results showed that 58.4% identified the Pollution Control Board of India as the regulatory body, 55.9% understood hospital waste handling, and 91.9% were aware of dental waste categories. While 89.8% supported practical training in dental schools, only 32.3% knew eco-friendly waste conversion methods. Postgraduates had better knowledge than students and interns, but overall awareness was insufficient, highlighting the need for specialized biomedical waste management training in clinical settings [32].

## Conclusions

The results reveal trends in BMWM awareness and behaviors, emphasizing the role of education in promoting responsible waste management practices. The study underscores the need for prioritizing BMWM training, particularly at the undergraduate level, to ensure consistent adherence to safety and disposal protocols in healthcare settings. 

Higher education levels were linked to better Biomedical Waste (BMW) management knowledge (p=0.001), with faculty scoring highest, followed by postgraduates and undergraduates (Table 5 (Tab. 5)). Analysis of mean scores showed that faculty scored the highest, while undergraduates had the lowest scores. This may suggest that increased knowledge in each group correlates with experience and practical application in daily activities.

Compared to other countries, India’s biomedical waste management (BMWM) regulations are relatively stringent, but enforcement and infrastructure remain areas of concern. The Biomedical Waste Management Rules 2016 in India set clear guidelines for the segregation, collection, and disposal of biomedical waste (BMW) (Ministry of Environment, Forest and Climate Change, 2016). However, the implementation is inconsistent across regions, particularly in smaller healthcare facilities and rural areas, where infrastructure and awareness are lacking [8], [33].

In developed countries like the U.S., Germany, and Japan, comprehensive BMWM systems have been in place for years. The U.S. follows the Resource Conservation and Recovery Act (RCRA), which regulates the treatment and disposal of hazardous waste, including biomedical waste (EPA, 2021) [34]. Biowaste from hospitals does not have to be sterilized in Germany, but must be disinfected or incinerated if it contains pathogens with the risk of transmission or further spread. This applies to brucellosis (blood), cholera (stool, vomit), diphtheria (sputum, pharyngeal secretions, wound secretions), meningitis, encephalitis (sputum, pharyngeal secretions, wound secretions), anthrax (sputum, pharyngeal secretions, wound secretions), paratyphoid A, B, C (stool, urine, bile, blood), plague (sputum, pharyngeal secretions, wound secretions), smallpox (all material coming from the patient), poliomyelitis (sputum, pharyngeal secretions, stool), dysentery, HUS (stool), SARS, COVID-19 (sputum, pharyngeal secretions), rabies (sputum, pharyngeal secretions), TSE, CJD, vCJD (blood, cerebrospinal fluid), active tuberculosis (sputum, urine, stool), tularemia (wound secretion, pus), typhus abdominalis (stool, urine, bile, blood), viral hemorrhagic fevers, including infections caused by hantaviruses (blood, sputum, pharyngeal secretions, wound secretions, urine), viral hepatitis A (stool), and viral hepatitis B/D, C, E (waste containing discharged blood or blood-contaminated fluids). Clinical waste such as disposable clothing, wound and plaster dressings, underwear, and diapers, as well as sharp and pointed waste, must be collected in carefully sealed containers, if necessary in combination with return containers, and transported to the central storage and transfer point. It does not have to be disinfected. Infectious material from microbiological laboratories must be disinfected within the laboratory building or collected in type-tested containers. If the presence of CJD and other spongiform encephalopathies is suspected, special attention must be paid to the disposal of autopsy material and similar; even small tissue residues must be incinerated. Additionally, countries like Sweden and Germany emphasize sustainability, using advanced technologies for disinfection, recycling, and waste-to-energy processes [35], [36], [37]. These nations also have rigorous monitoring and enforcement mechanisms and focus on reducing environmental impact by recycling non-hazardous waste, which is less common in India.

India, however, faces challenges with proper disposal and sterilization of BMW, with many healthcare facilities lacking the necessary infrastructure for technologies like autoclaving or incineration [38]. While the Indian government has improved regulatory frameworks, better enforcement, public education, and infrastructure investment are still needed to align with the global standards seen in developed nations.

### Limitations of the study 

In Biomedical Waste Management (BMWM) studies, cross-sectional designs, despite their limitations, offer several advantages. They provide a quick snapshot of current practices, which is useful for identifying prevalent issues and trends. With data collected at a single point, these studies are cost-effective and less time-consuming compared to longitudinal studies. Self-reported data can still offer valuable insights into participants' perceptions and self-assessed compliance. While non-random sampling may limit generalizability, it can still provide meaningful information within the specific context of the sample. Furthermore, cross-sectional studies can highlight areas where immediate interventions or improvements in education are needed, making them useful for policy makers and educators in the short term.

## Notes

### Competing interests

The authors declare that they have no competing interests.

### Ethical approval 

This was a questionnair- based study. A google form was created and those willing to participate were included the study. Anonymity was maintained throughout the study. The school of Dental Sciences, Manav Rachna International Institute of Research and Studies Institute’s ethics committee approved the study. 

### Funding

None. 

### Authors’ ORCIDs

- Mahesh S: https://orcid.org/0000-0002-7574-3103
- Hemalata K: https://orcid.org/0009-0004-1429-4860
- Shanta R: https://orcid.org/0000-0002-7975-3309
- Vashistha U: https://orcid.org/0000-0002-6780-1414
- Krishnakumar K: https://orcid.org/0009-0006-1201-5017
- Arora S: https://orcid.org/0009-0006-5803-5312
- Gupta A: https://orcid.org/0000-0001-8047-5054

## Figures and Tables

**Table 1:**
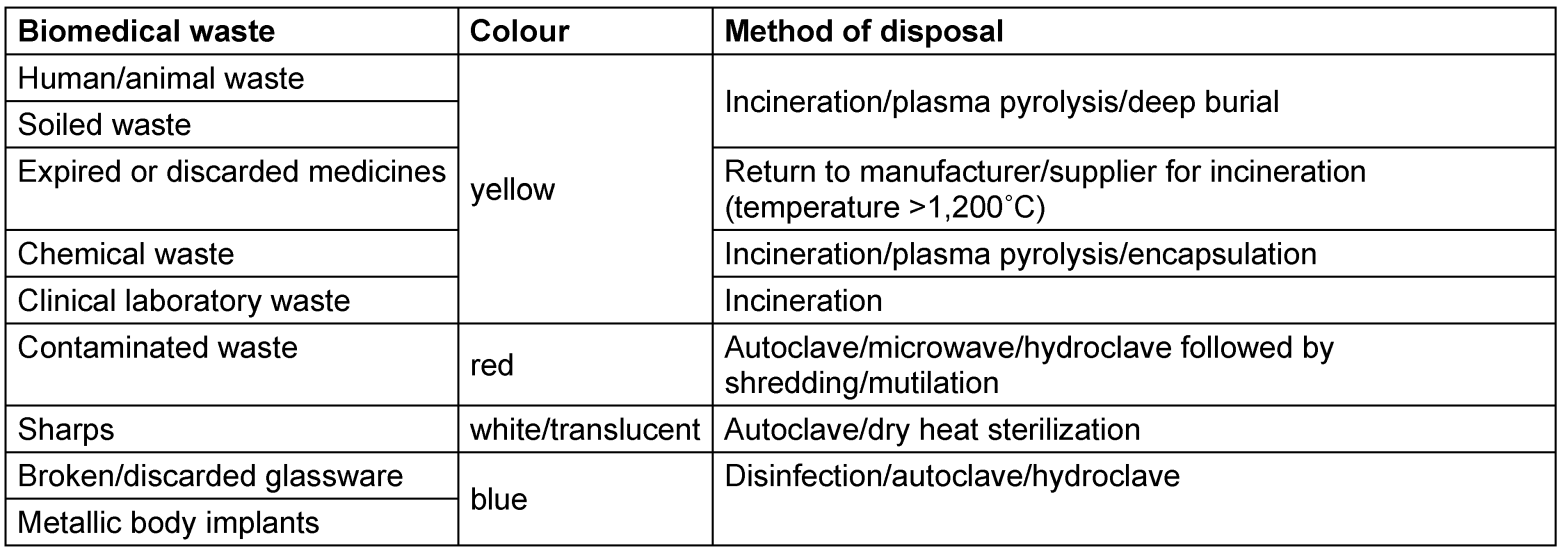
Biomedical wastes, categories, and treatment and disposal options (modified according to [8])

**Table 2:**
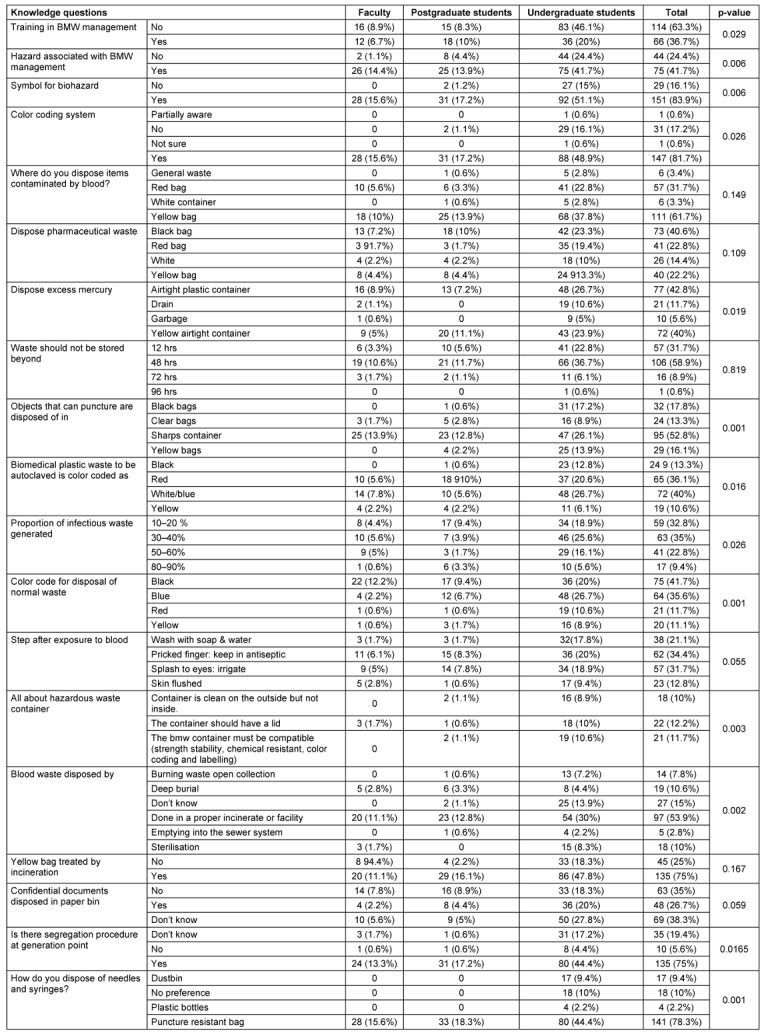
Response to knowledge questions based on education level

**Table 3:**
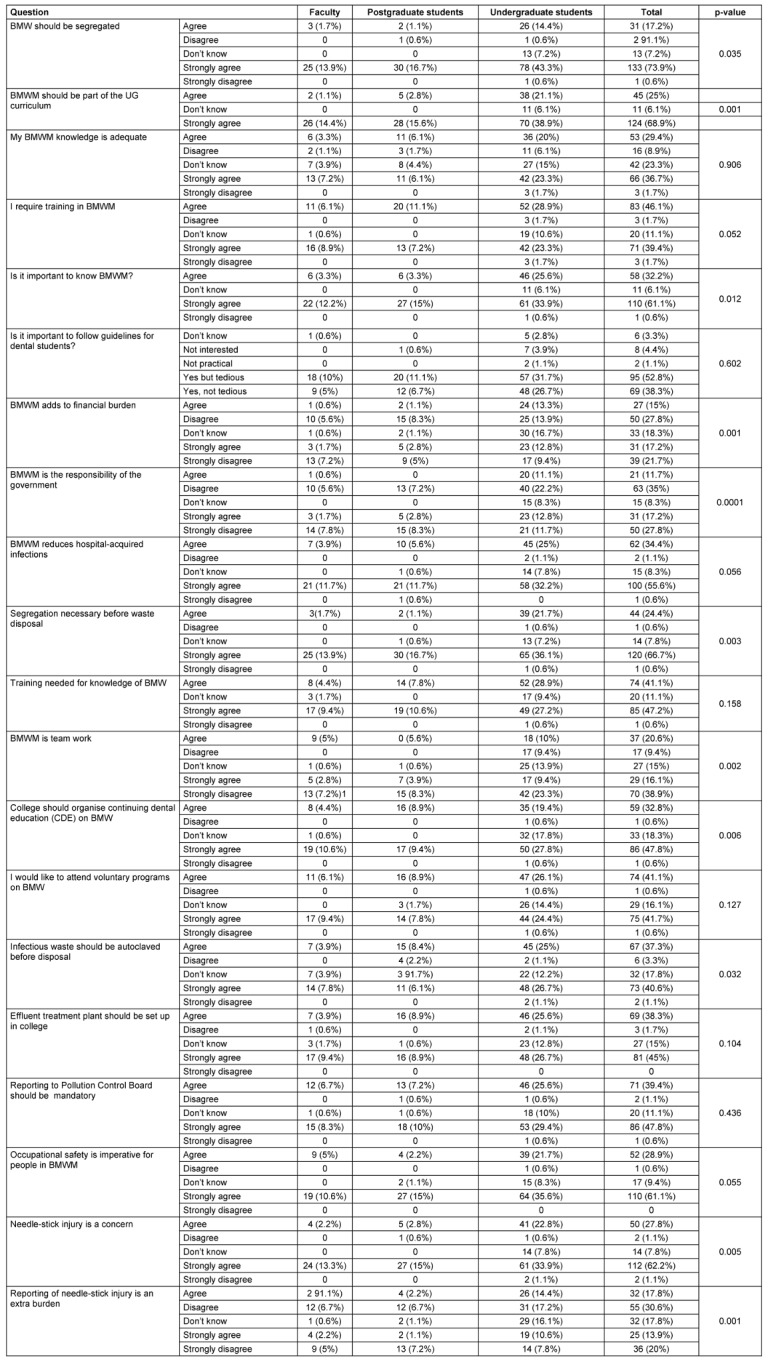
Response to attitude questions based on education level

**Table 4:**
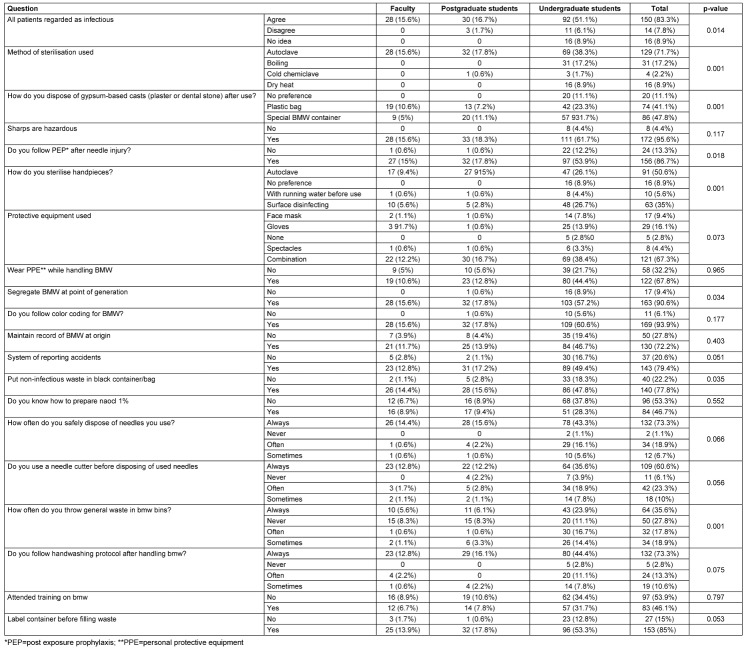
Response to practice questions based on education

**Table 5:**
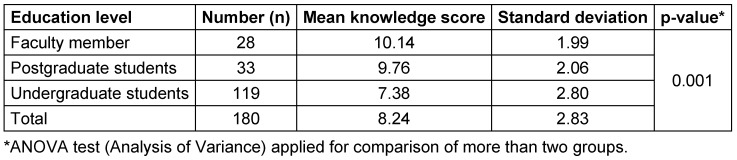
Mean knowledge score among the study population

## References

[1] Kapoor D, Nirola A, Kapoor V, Gambhir RS (2014). Knowledge and awareness regarding biomedical waste management in dental teaching institutions in India- A systematic review. J Clin Exp Dent.

[2] (2017). Report on health-care waste management (HCWM) status in Countries of the South-East Asia Region (SEA Region), April 2017.

[3] World Health Organization (2014). Safe management of wastes from health-care activities.

[4] Turnberg WL, Frost F (1990). Survey of occupational exposure of waste industry workers to infectious waste in Washington State. Am J Public Health.

[5] Farmer GM, Stankiewicz N, Michael B, Wojcik A, Lim Y, Ivkovic D, Rajakulendran J (1997). Audit of waste collected over one week from ten dental practices. A pilot study. Aust Dent J.

[6] Ozbek M, Sanin FD (2004). A study of the dental solid waste produced in a school of dentistry in Turkey. Waste Manag.

[7] Paryag A, Rafeek R, Pilgrim A (2010). Mercury pollution from dental amalgam waste in Trinidad and Tobago. J Water Res Protec.

[8] Ministry of Environment, Forest and Climate Change, Government of India (2016). Notification: Bio-Medical Waste Management Rules, Part II, Section 3, Sub-section (i).

[9] Centre for Chronic Disease Control, Centre for Environmental Health, Public Health Foundation of India, Health Care Without Harm (2016). Pictorial Guide on Biomedical Waste Management (BMWM) Rules, 2016 (amended in 2018 & 2019).

[10] Awodele O, Adewoye AA, Oparah AC (2016). Assessment of medical waste management in seven hospitals in Lagos, Nigeria. BMC Public Health.

[11] Kishore J, Goel P, Sagar B, Joshi TK (2000). Awareness about biomedical waste management and infection control among dentists of a teaching hospital in New Delhi, India. Indian J Dent Res.

[12] Sudhakar V, Chandrashekar J (2008). Dental health care waste disposal among private dental practices in Bangalore City, India. Int Dent J.

[13] Basavaraj TJ, Shashibhushan BL, Sreedevi A (2021). To assess the knowledge, attitude and practices in biomedical waste management among health care workers in dedicated COVID hospital in Bangalore. Egypt J Intern Med.

[14] Khubchandani K, Devi KM, Gunasekaran S, Yeturu SK, Ramanarayanan V (2020). Knowledge, attitude, and practices of biomedical waste management among clinical dental students. J Global Oral Health.

[15] Sharma A, Sharma V, Sharma S, Singh P (2013). Awareness of biomedical waste management among health care personnel in jaipur, India. Oral Health Dent Manag.

[16] Singh BP, Khan SA, Agrawal N, Siddharth R, Kumar L (2012). Current biomedical waste management practices and cross-infection control procedures of dentists in India. Int Dent J.

[17] Patnaik S, Sharma N (16). Assessment of cognizance and execution of biomedical waste management among health care personnel of a dental institution in Bhubaneswar. J. Indian Assoc. Public Health Dent.

[18] Soni D, Deepanker A, Saroshe S, Yuwane P, Singh A, Dixit S (2023). Perception and practice of hospital waste management among medical and nursing personnel. Int J Med Pharm Res.

[19] Indumathy M, Mukesh S (2021). Biomedical waste management − a review. Int J Soc Rehab.

[20] Mathur V, Dwivedi S, Hassan M, Misra R (2011). Knowledge, Attitude, and Practices about Biomedical Waste Management among Healthcare Personnel: A Cross-sectional Study. Indian J Community Med.

[21] Dixit AM, Bansal P, Jain P, Bajpai PK, Rath RS, Kharya P (2021). Assessment of biomedical waste management in health facilities of uttar pradesh: an observational study. Cureus.

[22] Hossain MS, Balakrishnan V, Rahman NN, Sarker MZ, Kadir MO (2012). Treatment of clinical solid waste using a steam autoclave as a possible alternative technology to incineration. Int J Environ Res Public Health.

[23] Ferdowsi A, Ferdosi M, Mehrani MJ (2013). Incineration or autoclave? A comparative study in isfahan hospitals waste management system (2010). Mater Sociomed.

[24] Patil GV, Pokhrel K (2005). Biomedical solid waste management in an Indian hospital: a case study. Waste Manag.

[25] Almuneef M, Memish ZA (2003). Effective medical waste management: it can be done. Am J Infect Control.

[26] Singh T, Ghimire TR, Agrawal SK (2018). Awareness of Biomedical Waste Management in Dental Students in Different Dental Colleges in Nepal. Biomed Res Int.

[27] Hossain MS, Santhanam A, Nik Norulaini NA, Omar AK (2011). Clinical solid waste management practices and its impact on human health and environment--A review. Waste Manag.

[28] Giusti L (2009). A review of waste management practices and their impact on human health. Waste Manag.

[29] Singh RD, Jurel SK, Tripathi S, Agrawal KK, Kumari R (2014). Mercury and other biomedical waste management practices among dental practitioners in India. Biomed Res Int.

[30] Udoye C, Aguwa E (2008). Amalgam safety and dentists' attitude: a survey among a Subpopulation of Nigerian dentists. Oper Dent.

[31] Mohan S, Priyank H, Kumar G, Viswanath B (2024). Knowledge, attitudes, and practices of undergraduate dental students about sterilization, disinfection, and infection control: a questionnaire-based study. Cureus.

[32] Puri S, Smriti K, Pentapati CK, Singh R, Vineetha R, Tamrakar A (2019). Assessment of awareness about various dental waste management practices among dental students and practicing clinicians. Pesqui Bras Odontoped Clín Integr.

[33] Dhole KS, Bahadure S, Bandre GR, Noman O (2024). Navigating challenges in biomedical waste management in India: a narrative review. Cureus.

[34] US Environmental Protection Agency (EPA) (2025). Biomedical Waste.

[35] Negrete-Cardoso M, Rosano-Ortega G, Álvarez-Aros EL, Tavera-Cortés ME, Vega-Lebrún CA, Sánchez-Ruíz FJ (2022). Circular economy strategy and waste management: a bibliometric analysis in its contribution to sustainable development, toward a post-COVID-19 era. Environ Sci Pollut Res Int.

[36] Hansen D, Mikloweit U, Ross B, Popp W (2014). Healthcare waste management in Germany. Int J Infect Control.

[37] Kramer A, Brill FHH (2024). Hygienic evaluation of the Resourcify GmbH concept for recovering raw materials from recyclable medical devices after surgery. GMS Hyg Infect Control.

[38] Datta P, Mohi GK, Chander J (2018). Biomedical waste management in India: Critical appraisal. J Lab Physicians.

